# Pathophysiology, Clinical Characteristics of Diabetic Cardiomyopathy: Therapeutic Potential of Natural Polyphenols

**DOI:** 10.3389/fnut.2020.564352

**Published:** 2020-12-03

**Authors:** Neha Atale, Dhananjay Yadav, Vibha Rani, Jun-O Jin

**Affiliations:** ^1^Jaypee Institute of Information Technology, Noida, India; ^2^Department of Medical Biotechnology, Yeungnam University, Gyeongsan, South Korea; ^3^Research Institute of Cell Culture, Yeungnam University, Gyeongsan, South Korea

**Keywords:** diabetic cardiomyopathy, oxidative stress, extracellular matrix, cardiac hypertrophy, natural polyphenols

## Abstract

Diabetic cardiomyopathy (DCM) is an outcome of disturbances in metabolic activities through oxidative stress, local inflammation, and fibrosis, as well as a prime cause of fatality worldwide. Cardiovascular disorders in diabetic individuals have become a challenge in diagnosis and formulation of treatment prototype. It is necessary to have a better understanding of cellular pathophysiology that reveal the therapeutic targets and prevent the progression of cardiovascular diseases due to hyperglycemia. Critical changes in levels of collagen and integrin have been observed in the extracellular matrix of heart, which was responsible for cardiac remodeling in diabetic patients. This review explored the understanding of the mechanisms of how the phytochemicals provide cardioprotection under diabetes along with the caveats and provide future perspectives on these agents as prototypes for the development of drugs for managing DCM. Thus, here we summarized the effect of various plant extracts and natural polyphenols tested in preclinical and cell culture models of diabetic cardiomyopathy. Further, the potential use of selected polyphenols that improved the therapeutic efficacy against diabetic cardiomyopathy is also illustrated.

## Introduction

Diabetes, a group of metabolic diseases associated with damage and dysfunction of various organs, especially the heart, and lead to cardiovascular diseases. The current burden of diabetes has become a major threat to the health of populations, which reflects the cumulative effects of risk factors over the life span of people. Present western lifestyle and environmental factors promote the progression of these pathological conditions and are held responsible for rising rate of diabetic cardiomyopathies (1, 2).

Persistent hyperglycemia causes the structural and molecular changes in cardiomyocytes, by increased production of advanced glycation end products (AGEs) in diabetes due to constant oxidative stress (3, 4). AGEs accumulate in various tissues and can link with other proteins such as collagen type IV, laminin, and fibronectin, resulting in impaired cardiac function and enhanced myocardial stiffness. A significant increase in AGEs and its specific receptors (RAGEs) on cells trigger oxidative stress and activate protein kinase C, finally disrupting cellular and molecular functions (5, 6). Oxidative stress induces the breaks in DNA, resulting in increased activity of poly ADP ribose polymerase (PARP) enzyme, which further promote the progression of various cardiovascular diseases (7).

The preponderance of diabetes will be accelerated by 50–60% in the population from 2015 to 2030, and also annual mortality rate attributing diabetes will be increased by 38% in the US population. Global healthcare expenditures were found to be higher to treat and prevent diabetes and its associated complications (~376 billion US Dollars (USD) in 2010). This cost is projected to exceed ~622 billion USD by 2030 (8, 9). However, the outcome of such expenditures is not satisfactory and people of low-income countries are still devoid of the treatments. Hence, an affordable therapy is required against diabetes, especially in the poorer socio-economic sections of country worldwide.

An alternative therapy by using plant polyphenols may have a great choice to end the cardiovascular complications developed by DCM. In this review, we discusses the pathophysiology and clinical features of DCM and the possible role of polyphenol in relation to the DCM therapy has been explored.

## Pathophysiology of Diabetic Cardiomyopathy

**Figure 1:**
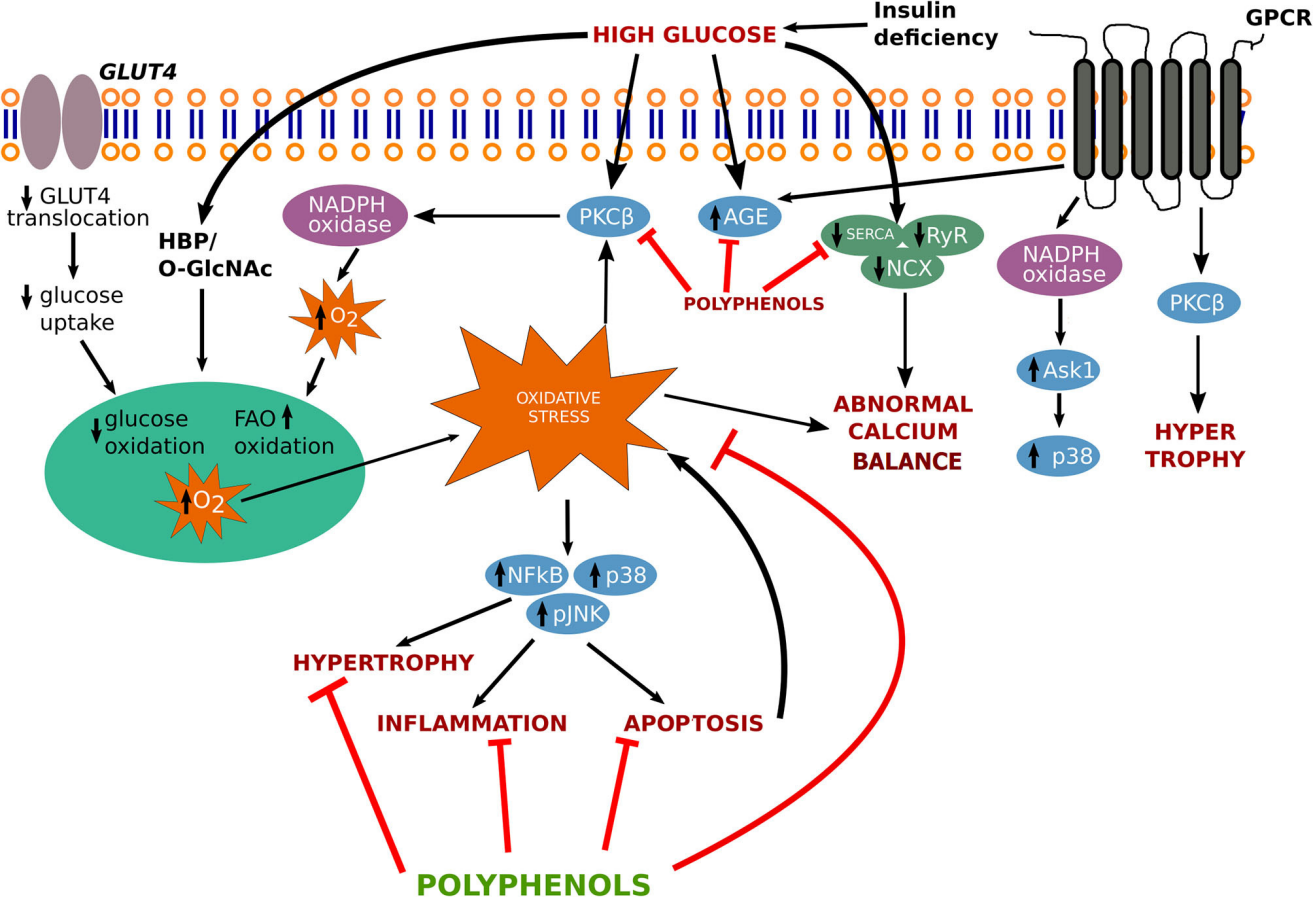
Signaling pathway involved in the progression of diabetic cardiomyopathy. AGEs, advanced glycation end-products; ASK1, apoptotic signal regulating kinase-1; GLUT-4, glucose transporter-4; GPCR, G protein coupled receptor; HBP, hexosamine biosynthesis pathway; JNK, c-Jun N-terminal kinase; NADPH, nicotinamide adenine dinucleotide phosphate; NCX, sodium–calcium exchanger; O-GlcNAc, O-linked *beta-N-acetylglucosamine; p38, p38 MAPK; PKC*β*, protein kinase C-*β*; RyR, ryanodine receptor SERCA, sarcoplasmic reticulum Ca2*+ *ATPase; NF-*κ*B, nuclear factor-* κ*B*.

In the following sections, we have described the physiological mechanisms associated with progression of diabetic cardiomyopathy (Figure 1).

### Oxidative Stress: Critical Contributor to Diabetic Cardiomyopathy

Free radicals are generated due to the continuous production of the oxygen, and considered as “reactive oxygen species” (ROS) (10). Stress inducing agents stimulate the drastic release of these oxidants and create an imbalance in the equilibrium of ROS production and antioxidant capability (11, 12).

Endothelial nitric oxide synthase (eNOS), a critical enzyme of endothelial cells, produces NO· and gets altered due to high glucose stress, which ultimately leads to vascular endothelial cell dysfunction. NO· further produces ONOO–, a cytotoxic free radical responsible for disturbing cardiovascular function (13). Oxidative stress is determined by the overproduction of ROS and RNS molecules. The unstable configuration leads to the breakdown of lipids, inactivation of enzymes, cell membranes, and DNA damage.

### Calcium Homeostasis

Intracellular calcium (Ca^2+^) is a significant marker of cardiac contraction. Hyperglycemic stress enhances Ca^2+^ accumulation in cardiac myocytes, which imposes impairment in the ionotropic response in the heart. Ca^2+^ influx activates its release after binding to troponin C and generates tension by activating the sliding of thin and thick filaments, which further resulting in cardiac contraction (14). Intranuclear Ca^2+^ alteration may also change various kinases activities namely (extracellular-signal-regulated kinase) ERK, (microtubule-associated protein kinase) (MAPK) and Janus kinase (JNK). MAPKs are also involved in the transcription of *c-fos* and *c-jun*, which activate phospholipase A_2_, resulting in the plasma membrane and intracellular membranes permeabilization, further leading to cell death (15) (Figure 1).

### Rennin-Angiotensin System

In diabetic cardiomyopathy, rennin-angiotensin (RAAS) system is considered a life-saving system. RAAS is a complex pathway whose activation triggers a cascade of events leading to cardiovascular disease. Studies show that infusing Angiotensin-II (AngII) leads to stimulation of ACE/AngII/AT1R complex accelerating atherosclerosis and blocking of RAAS protect against cardiac damage (16). RAAS can be activated by hyperglycemia, leading to production of Ang-II. It has been known that the Ang-II can produce ROS through NADH/NADPH oxidase system. RAAS's role in NADH/NADPH bound oxidase is further supported by studies showing the effectiveness of ramipril (and ACE inhibitor) in preventing upregulation of p47phox, p22phox, and reduced NADH driven oxide production (17). This led to reduced fibrosis and hypertrophic gene expression. Blocking of Ang-II also showed reduced expression of p22phox, NADH-oxidases and HG-induced p47phox (18). These studies show that the RAAS activity in diabetes supports an interaction between Ang-II and NADPH-oxidases in cardiomyocyte.

## Extracellular (ECM) Remodeling in Diabetic Cardiomyopathy

The extracellular matrix is a complex meshwork of fibers comprised of proteins, polysaccharides, and provides structural as well as functional support to the surrounding cells, which are important for the cell-to-cell communication and adhesion. Alteration in the extracellular matrix components contributes to diabetic cardiomyopathy, which enhances stress in the diabetic heart which involves changes in mass, shape, and volume of the left ventricle, leading to ischemia, and pressure overload (19, 20). Changes in physiological conditions due to stress stimulus can trigger various proteases activities such as serine proteases and matrix metalloproteinases (MMPs) that cause alteration in the expression of collagen, fibronectin, and ultimately leads to ECM remodeling (21).

Collagen fibrils are the fundamental blocks of extracellular matrix and give mechanical strength, stiffness, and toughness to the vasculature (22). The extracellular collagen matrix of the myocardium has a major function in maintaining cardiac organization. An excessive accumulation of fibrillar collagen in the myocardium was reported in hypertrophied heart (23). In absence of secondary risk factors such as hypertension or coronary artery disease, cardiac dysfunction in diabetic patients, increased collagen I, III, and IV deposition has been found to results in fibrosis and poor LV function (24). There is a delicate balance between continual degradation and synthesis of collagen in ECM. Specific collagen degrading MMPs enzymes as well as their inhibitors (tissue inhibitors of metalloproteinases, TIMPs), are essential in collagen remodeling (25).

### Matrix Metalloproteinases: Key Enzymes for ECM Modulation

MMPs have several conserved domains with different substrate specificity and inducibility. They play a major function in wound healing, tissue repair, and remodeling in various diseases. MMPs are of two types, membrane-bound and secretory. Membrane-type MMPs (MT-MMPs) work in close proximity with the cell, whereas the secreted MMPs act within the matrix, away from the cells from which they are synthesized. MMP-2 (Gelatinase A) and MMP-9 (Gelatinase B) are the most abundant secreted proteases, which degrade gelatin. They are categorized in 6 main classes such as- gelatinases, collagenases, stromelysins, matrilysins, membrane-type MMPs (MT-MMPs), and other MMPs (26).

MMPs are regulated in expression or activity and cellular inhibition by endogenous tissue inhibitors of metalloproteinases (TIMPs) (27). The MMP-TIMP balance maintains the integrity of ECM by regulating the debasement rate of ECM proteins and tissue remodeling. Most of MMP inhibitors have wide-spectrum actions on other MMPs and could cause adverse effects. Usage of synthetic MMP inhibitors in experimental animal models against upregulated activities of MMPs are failed in clinical trials, except FDA approved doxycyclin. The synthesis and design of new generation biological and synthetic MMP inhibitors are required.

## Cardiac Hypertrophy in Diabetic Cardiomyopathy

High glucose uptake initiates an imbalance in myocardial energetics and results in myocardial ischemia or hypertrophy. Whenever cells are exposed to high glucose stress, they enlarge and undergo hypertrophy (increase in size, not in number) to combat the excessive stress, resulting in an increase in myocytes length (eccentric hypertrophy), or myocytes width (concentric hypertrophy*)*, which further enhances thickening of the septum and ventricular wall (28).

Cardiac hypertrophy, a phenomenon observed with many forms of human heart disease including diabetic cardiomyopathy, results in an increase in protein synthesis, addition of sarcomeres and fetal genes re-expression such as myosin heavy chain (β-MHC) and GATA-1 and activation of early response genes, such as c-jun, c-fos, and c-myc etc. In hypertrophic conditions, various signaling pathways such as mitogen-activated protein kinases, tyrosine kinase Src, GTP-binding protein Ras, protein kinase C, phosphoinositol 3-kinase are involved (29) (Figure 1). Transforming growth factor β (TGF-β) mediates the production of transcription factors such as nuclear factor kappa B (NF-kB), small mothers against decapentaplegic (SMAD), signal transducer activating protein-1 (AP-1), and activator of transcription (STAT) that takes part in MMPs and TIMPs transcription leading to remodeling of the extracellular matrix.

Cardiac hypertrophy is prevalent in asymptomatic type 2 diabetes patients (30). The following fetal genes are used as an indicator/marker of cardiac hypertrophy under diabetic stress.

### Sarcoplasmic Reticulum Ca^2+^ ATPase 2 (Serca2)

Sarcoplasmic reticulum Ca2+ ATPase 2 (Serca2), a candidate molecule for re-uptake of calcium into the sarcoplasmic reticulum, allows the muscle relaxation. Decreased Serca2 expression level was found in the diabetic heart and undergone diastolic dysfunction in cardiomyopathy (31). The SR Ca^2+^ loading determines the Ca^2+^ ion concentration available for next contraction dictating the rate of myocardial relaxation (32). Increases in Serca2 activity tend to enhance myocardial contraction and relaxation.

### Myofilament Proteins

Myosin, actin, and titin filament proteins are highly expressed in the fetal heart than that of adult and major signs of cardiac hypertrophy. Myosin-binding protein C, interacts with actin, and changes the myosin cross-bridges (33) Myosin heavy chains (MHC), with integral ATPase activity, is one of the most underlying ways to find changes of MHC composition in the hypertrophied heart. Z-disc MLP–TCAP–titin complex defects can lead to cardiomyopathy and heart failure development.

### Peptide Hormones

Atrial and brain natriuretic peptide (ANP and BNP) are small hormones, and their secretion occurs during cardiac stress. For acute heart failures, ANP and BNP can be clinically administered as therapeutic agents. For chronic cases, on the other hand, neprilysin (responsible for the degradation of ANP and BNP) (34) inhibitor is used. Recent studies reported that human proBNP, in bloodstream and its post- translational modification at the N-terminal region could lead to its higher levels in cardiac patients (35).

### Transcription Factors and Inflammatory Signals

The main transcription factor, critically involved in hypertrophy is GATA-4, which was found to be highly expressed in the fetal myocardium. It triggers a wide group of heart- specific genes such as α and β-MHC (Myosin heavy chain), MEF-2 (Myocyte enhancer factor-2), SP-1 (Specificity protein-1), and NFκB (Nuclear factor-κappa B) associated with hypertrophy (36).

## Natural Polyphenols: Therapeutics of DCM

Natural products have multiple pharmacological actions against pathological conditions and the use of these products is safer than synthetic drugs. The use of plants as a source of natural polyphenols in various forms of traditional medicines from ancient time. Huynh et al. illustrates the pathway involving several cascades involves in the progression of diabetic cardiomyopathy (37). Based on the findings the key findings on disease pathways and treatment options such as targeting antioxidant-signaling pathways, we also proposed a figure that involves in the treatment strategy through various polyphenolics to target several genes and ameliorates the pathological conditions arises by cardiomyopathy (Figure 1).

Herbal remedies are gaining significant attention and that nearly 80% of the total world population uses conventional medicine, comprising 40,000–70,000 medicinal plants (38, 39). In recent years, due to toxicological concerns with the synthetic substances in food and increasing awareness about herbal therapies, the use of natural substances are demanding. Herbal formulations are found to be cost-effective and having low side effects. Plant extracts have recently gained interest due to their antiglycoxidative activities, that defend cells against the free radical attack and have numerous biological consequences (40, 41). The synthesis of different organic solvent extracts may differ in quality and concentration depending upon the difference in the polarity of the solvent used for extraction and the extracted polyphenols (42, 43). Phenolic compounds such as gallic acid, caffeic acid, ferulic acid, trans-resveratrol, quercetin, fisetin have been shown to act as natural antioxidants by neutralizing free radicals (44, 45).

We have summarized below some plant extracts and natural polyphenols, showing doses of polyphenols, experimental models studied, and key findings, which further suggest their beneficial effects against cardiovascular abnormalities (Table 1). *In vivo* studies indicated the beneficial effects of some polyphenols in DCM such as methanolic extract of *S. cumini* seeds treated group showed normal morphological cardiac features compared to the diabetic control. This may be due to the presence of quercetin, ellagic acid, rutin, and gallic acid in the extract which contributes to reducing aspartate aminotransferase (AST), creatine kinase-MB (CK-MB), and lactate dehydrogenase (LDH) level up to the normal (46, 47). The combined effect of A. sativum and voluntary exercise has worked as a powerful defense system, which decreases HbA1c and malondialdehyde (MDA) production in cardiomyocytes of diabetic models (48). *B. oleracea* also found to upregulate Nrf2 activation, a critical marker of DCM, which were found to be decreased in diabetic models (49). Decreasing MDA and AGEs formation levels was observed on the administration of *H. sabdariffa* (100 mg/Kg) in STZ treated rats, which also improved cardiac function by augmenting mitochondrial antioxidant defense (50). Administration of *M. oleifera* significantly reduced the lipid peroxidation products and increased the enzymatic as well as non-enzymatic antioxidants in the diabetic rat (51). Administration of A. augusta and A. marmelos could significantly reduce the levels of Interleukin (IL-6) IL-6, IL-1β, and (tumor necrosis factor- α) TNF-α in cardiac tissues of diabetic rats (52, 53), stimulates the antioxidants defense system by increasing catalase (CAT), superoxide dismutase (SOD) activities, and maintained the cardiac integrity. *E. oleracea* treatment along with exercise reduced leptin, IL-6, and TNF-α serum levels in diabetic models, which may improve the insulin sensitivity (54). *P. pinnata* significantly diminished the activity of cardiac enzymes such as LDH, CK-MB, AST compared with diabetic rats showing its cardioprotective effects (55). Resveratrol also promotes Nrf2 mediated cardiac protection (56). Treatment of catechins significantly increases SOD, CAT, and GSH activities up to control in diabetic rat hearts, however decreasing the higher levels of cardiac biomarkers CK-MB, AST, LDH, and troponin T, suggesting its cardioprotective effect (57). Curcumin supplementation leads to lowering the level of blood glucose and proinflammatory cytokines in DCM models (58).

**Table 1:** Studies of some polyphenols in *in vivo* and *in vitro* diabetic models.

**No**.	**Plant extracts/Polyphenols**	**Experimental model/cells**	**Polyphenols dose**	**Key findings**	**References**
1	*Syzigium cumini*	STZ induced diabetic rats Alloxan induced diabetic rats H9C2 cardiomyocytes	50 mg/Kg 150 mg/Kg 9 μg/ml	↓ fasting glucose levels ↓ LDH, CKMB, and AST ↓ TNF-α and IL-6 ↓ CAT and SOD	(47) (46) (59, 60)
2	*Allium sativum*	STZ induced diabetic rats H9C2 cardiomyocytes	500 mg/Kg 0.25 mg/ml	↓ Blood glucose, Hemoglobin A1c and MDA ↓Na+/K+-ATPase protein level	(48) (61)
3	*Brassica oleracea*	T2DM rats H9C2 cardiomyocytes	0.5 and 1.0 mg/Kg 100, 200, and 300 μg/ml	↑ NrF2 activation	(49) (62)
4	*Hibiscus sabdariffa*	STZ induced diabetic rats	100 mg/Kg	↑ CAT, SOD and GSH Improve cardiac contraction and relaxation	(50)
5	*Moringa oleifera*	STZ induced diabetic rats	300 mg/Kg	↓Serum glucose ↓Hemoglobin a1c ↑ CAT, SOD and GPx	(51)
6	*Abroma augusta*	STZ induced diabetic rats	100 and 200 mg/Kg	↑ CAT, SOD and GSH ↓cholesterol, LDH	(53)
7	*Aegle marmelos*	Alloxan induced diabetic rats	200mg/Kg	↓TBARS and LDH ↑ CAT, SOD and GPx	(52)
8	*Eutropea oleracea*	STZ induced diabetic rats	200 mg/Kg	↑pAKT, IL-6 ↑ GLP ↑Adiponectin	(54)
9	*Pongamia pinnata*	STZ induced diabetic rats	100 mg/Kg	↓LDH, CKMB, and AST	(55)
10	*Resveratrol*	STZ induced diabetic rats H9C2 cardiomyocytes	10 mg/Kg 25 μM	↑NrF2 levels Modulates Autophagy	(56) (63)
11	*Catechin* (EGCG)	STZ induced diabetic rats H9C2 cardiomyocytes	2 mg/Kg 50 and 100 mg/l	↑ CAT, SOD, and glutathione ↓ IL-1 β, IL-6, and TNF-α adhesion molecules Inhibited telomere attrition, telomere repeat-binding factor-2 loss and p53	(57) (64)
12	*Curcumin*	STZ induced diabetic rats H9C2 cardiomyocytes	(200 mg/kg) 8 μM	↓ IL-6 and TNF-α levels Prevent the nuclear localization of GATA-4 ↓β-myosin heavy chain expression ↓ Reduced matrix metalloproteinase-9 levels ↓ Apoptosis, ROS, NADPH oxidase, carbonylation ↓ Bax, ↑Bcl-2, COX, SOD	(58) (65) (66) (67)

*STZ, streptozotocin; TNF-α, tumor necrosis factor-α; IL-1, interleukin-1; IL-6, interleukin-6; Nrf2, Nuclear factor erythroid 2-related factor 2; AST, aspartate aminotransferase; CAT, catalase; SOD, Superoxide dismutase; GSH, Glutathione; LDH, lactate dehydrogenase; AKT (Protein kinase B); TBARS (Thiobarbituric acid reactive substances)*.

Plant polyphenols have been studied extensively through *in vitro* cell models for the treatment of cardiovascular diseases. In cardiac H9C2 cells, gallic acid was found to suppress hypertrophy and fibrosis by regulating JNK2 signaling and Smad3 binding. In these cells, ferulic acid was also shown to protect cardiomyoblasts from high glucose induced oxidative stress by mediating Ca^2+^ homeostasis (68). Similarly, epigallocatechin-3-gallate mediated cardioprotection was observed through Akt/GSK-3β/caveolin signaling (69). Quercetin, a flavonoid, inhibits AP-1, and activates the PPAR-γ pathways leading to protection against cardiac hypertrophy (70). Another agents such as, resveratrol and kaempferol have been shown to involve in Sirt-1 dependent pathways to provide protection against ER stress and reoxygenation injuries (71, 72) leading to decrease in cardiomyocyte apoptosis. Administration of curcumin (10 μM) in cardiac cells, combat the glucose induced stress by reducing the overproduction of ROS and apoptosis through PI3K/Akt pathway (67).

Polyphenols are large phytochemicals and only a small fraction of them seem to be absorbed by the gastrointestinal tract in their consumed form (73, 74). It is assumed that a large portion of these is first decomposed by the gut microbiome into lower molecule metabolites that are then absorbed in the gut. The polyphenols could work in two ways: firstly, the polyphenols could be broken down in the gut to produce several different metabolites which are absorbed and enter the system circulation. Secondly, polyphenols can directly affect the gut microbiota leading to health benefits in the patients. This could be through a change in microbiome composition (75), by facilitating the generation of short-chain fatty acids (76) and improving oxygen levels by reacting with ROS and improving the immune system in the gut (77), however, such mechanisms are unavailable to *in vitro* models, which could be a major limitation of these studies.

## Conclusion and Future Perspectives

Hyperglycemia is strongly correlated with the manifestation of cardiac malfunction and heart failure. Preclinical studies also revealed the beneficial effects of antioxidants, anti-inflammatory agents on cardiac dysfunction.

Although DCM has been found in both type-1 and type-2 diabetes, hyperglycemia induced cardiac fibrosis has been mainly observed in type-I DM hearts. Type-2 diabetes is primarily associated with cardiomyocyte hypertrophy and steatosis (78–80). Therefore, the studies showed in this paper are limited for type 1 diabetes.

*In vitro* studies involving high dosage of anti-oxidants have shown their protective effects. However, larger studies especially *in vivo* and clinical trials have shown variable results. Large clinical trials with anti-oxidant agents like vitamin C and E have not been able to provide clear evidence of their beneficial effects in diabetic patients. One of the reason anti-oxidant treatments are failed because the overall oxidation levels in cells are strongly regulated. Simply using anti-oxidants concentrates, as supplements may not work, as intended and new techniques need to be developed to improve the efficacy of anti-oxidant treatments. One such recent successful method is by reversing loss of enzyme functions such as treatment with drugs that prevent ROS-induced eNOS uncoupling (81). Another approach could be delivery of anti-oxidant enzymes using viral transfection gene therapy and targeted delivery at cellular or sub-cellular levels (82). Additionally, nanoparticles based drug delivery may lead to an effective treatment against such diabetic cardiomyopathies.

## Author Contributions

NA and VR designed the manuscript. NA, DY, VR, and J-OJ wrote the manuscript. NA, DY, and J-OJ edited the manuscript. All authors contributed to the article and approved the submitted version.

## Conflict of Interest

The authors declare that the research was conducted in the absence of any commercial or financial relationships that could be construed as a potential conflict of interest.
